# Transcriptome analyses provide insights into the difference of alkaloids biosynthesis in the Chinese goldthread (*Coptis chinensis* Franch.) from different biotopes

**DOI:** 10.7717/peerj.3303

**Published:** 2017-05-18

**Authors:** Hanting Chen, Cao Deng, Hu Nie, Gang Fan, Yang He

**Affiliations:** 1 State Key Laboratory Breeding Base of Systematic Research, Development and Utilization of Chinese Medicine Resources, Chengdu University of Traditional Chinese Medicine, Chengdu, Sichuan, China; 2 DNA Stories Bioinformatics Center, Chengdu, Sichuan, China

**Keywords:** *Coptis chinensis* Franch., Transcriptome, RNA sequencing, Alkaloids biosynthesis

## Abstract

*Coptis chinensis* Franch., the Chinese goldthread (‘Weilian’ in Chinese), one of the most important medicinal plants from the family Ranunculaceae, and its rhizome has been widely used in Traditional Chinese Medicine for centuries. Here, we analyzed the chemical components and the transcriptome of the Chinese goldthread from three biotopes, including Zhenping, Zunyi and Shizhu. We built comprehensive, high-quality de novo transcriptome assemblies of the Chinese goldthread from short-read RNA-Sequencing data, obtaining 155,710 transcripts and 56,071 unigenes. More than 98.39% and 95.97% of core eukaryotic genes were found in the transcripts and unigenes respectively, indicating that this unigene set capture the majority of the coding genes. A total of 520,462, 493,718, and 507,247 heterozygous SNPs were identified in the three accessions from Zhenping, Zunyi, and Shizhu respectively, indicating high polymorphism in coding regions of the Chinese goldthread (∼1%). Chemical analyses of the rhizome identified six major components, including berberine, palmatine, coptisine, epiberberine, columbamine, and jatrorrhizine. Berberine has the highest concentrations, followed by coptisine, palmatine, and epiberberine sequentially for all the three accessions. The drug quality of the accession from Shizhu may be the highest among these accessions. Differential analyses of the transcriptome identified four pivotal candidate enzymes, including aspartate aminotransferaseprotein, polyphenol oxidase, primary-amine oxidase, and tyrosine decarboxylase, were significantly differentially expressed and may be responsible for the difference of alkaloids contents in the accessions from different biotopes.

## Introduction

*Coptis chinensis* Franch., the Chinese goldthread (‘Weilian’ in Chinese), one of the most important medicinal plants from the family Ranunculaceae, is native to China and has been widely used in Traditional Chinese Medicine (TCM) for centuries (Kamath, Skeels & Pai, 2009; Xiang et al., 2016). The major active components of the Chinese goldthread are protoberberine alkaloids (Kamath, Skeels & Pai, 2009), such as berberine, palmatine, jatrorrhizine, coptisine, columbamine, and epiberberine, have the efficacy of antiviral activity, anti-inflammatory, antimicrobial activity, dispelling dampness, removing toxicosis and detoxification (Tjong et al., 2011; Cui et al., 2016; Friedemann et al., 2016; Kim et al., 2016).

Despite the prominent roles of the Chinese goldthread in medicine, our understanding of its metabolic pathways of active alkaloids components is limited by a lack of genomic resources. In recent years, a number of studies focused on effective separation methods and the effectiveness of alkaloids and therapy against various diseases (Tjong et al., 2011; Cui et al., 2016; Friedemann et al., 2016; Kim et al., 2016; Zhang et al., 2011). However, the identification, purification and characterization of functional genes involved in metabolic pathways of active alkaloids components has received less attention, and only limited number of genes, such as (*S*)-Tetrahydroberberine oxidase (*(S)-THBO*) (Okada et al., 1988) and (*S*)-norcoclaurine synthase (*(S)-NCS*) (Bonamore et al., 2010) that involved in the biosynthetic pathway of berberine, has been investigated and reported. Moreover, although the distribution of the Chinese goldthread is limited to narrow regions west of China, such as Chongqing, Shaanxi and Guizhou province, due to variants of climate, topography and other environmental factors, the chemical composition of the alkaloids and the quantity of each alkaloids component in different biotope is different, resulting in different drug efficacy (Geng et al., 2010).

Deciphering genomes of medicinal species is of great importance in understanding and improving these poorly investigated species and will enable insights into the biochemistry and evolution of genes responsible for secondary metabolism biosynthetic pathways (Chen et al., 2012; Zhu et al., 2015; Yan et al., 2015; He et al., 2016a; Kellner et al., 2015). A pilot project on whole genome sequencing of the Chinese goldthread revealed that its genome size is nearly 1,116 Mb and the heterozygosity rate is as high as ∼1.1% (Sun et al., 2014), suggesting that producing a draft genome is not feasible if only using short read Illumina sequencing technology. More sophisticated method and platforms, such as combining with BAC library sequencing and/or third-generation sequencing platforms, maybe achieve a usable assembly version, but this is prohibitive to small research communities due to the expense, time, and expertise required. Fortunately, another promising technology is whole transcriptome shotgun sequencing (also called RNA Sequencing, RNA-Seq). High-quality transcriptome data would facilitate researches of functional genes and is also valuable for comparative biology studies. By using short paired-end reads, RNA-Seq has been proved to be an effective tool for transcripts reconstruction and quantification in a large variety of species (He et al., 2016b; Yang et al., 2014).

The specific goals of this study are to: (1) generate high-quality transcripts and unigenes sequences for the Chinese goldthread, which will provide reference transcriptome for further analyses; (2) identify pivotal candidate genes responsible for active metabolites biosynthesis in the Chinese goldthread. To set up the foundation for genomic studies of the Chinese goldthread, we sequenced its transcriptome using RNA-Seq. Based on the content of active components in producing areas*,* we analysed the active metabolites biosynthesis and explored pivotal candidate genes responsible for active metabolites biosynthesis.

## Materials and Methods

### Plant materials

The Chinese goldthread accession T01 was collected in Zhenping, Shaanxi, China; accession T02 was collected from Zunyi, Guizhou, China; and accession T03 was collected in Shizhu, Chongqing, China. Among them, only T03 was collected from genuine producing area. Whole plant of each accession was harvested and all samples were immediately frozen in liquid nitrogen and stored at −80 °C for later use.

### Detection of alkaloids by HPLC

HPLC analysis was performed on an Agilent 1200 Series LC system (Agilent Technologies, Ratingen, Germany), including a quaternary pump, an on-line degasser, a diode array detector, an auto-sampler and a column compartment. The chromatographic separation was performed on an Xtimate C18 column (4.6 mm × 250 mm, 5 µm). The mobile phase consisted of acetonitrile (A) and 30 mmol/L ammonium bicarbonate buffer containing 0.7% (v/v) ammoniae aqua and 0.1% (v/v) triethylamine (B). The gradient elution program was as follows: 10–25% A at 0–15 min, 25–30% A at 15–25 min, and 30–45% A at 25–40 min. The flow rate was kept at 1.0 mL/min. Column temperature was kept constant at 30 °C, and the injection volume was 10 µL. The detection wavelength was set at 270 nm. Sample preparation was performed according to our previously reported method (Fan et al., 2012). The dried powders of the Chinese goldthread samples (0.1 g) were accurately weighed into a clean Erlenmeyer flask, and macerated in 50 mL of hydrochloric acid-methanol solution (1:100, v/v). The sample was extracted for 30 min in an ultrasonic bath at room temperature and the loss of weight due to evaporation of solvent was replenished with hydrochloric acid-methanol solution (1:100, v/v). The sample solutions were filtered through a 0.45 µm membrane filter before HPLC analysis. The concentrations of berberine, palmatine, coptisine, epiberberine, columbamine, and jatrorrhizine in each sample with three replications were determined by using established calibration curves. The concentration (mg/g) is defined as the milligram of alkaloid per one gram dried tissue. For each kind of alkaloid, statistical analysis on one-way analysis of variance (ANOVA) was performed using Microcal Origin software (version 8.0).

### RNA isolation, cDNA library preparation and Illumina sequencing

Total RNA of the three samples was isolated from three accessions respectively using a modified CTAB method and purified by Micropoly (A) PuristTM mRNA purification kit (Ambion, Austin, TX, USA) according to manuals. The purity, concentration and integrity of the RNA were verified using NanoDrop 1000 spectrophotometer, Qubit 2.0 fluorometer and Agilent 2100 Bioanalyzer, respectively. Then, the mRNA was isolated using oligo (dT) magnetic beads and broken into fragments with a certain amount of Fragmentation Buffer. Fragments were used as templates to produce the first strand cDNA synthesis using random hexamers-primer. Buffer, dNTPs, RNaseH and DNA polymerase I were next added into the mixture to produce the second strand cDNA synthesis. The mixed cDNA was purified with AMPure XP beads. Then, ends of the purified cDNA were repaired and poly (A) was added. This was followed by the selection of suitable fragments using AMPure XP beads. PCR amplifications of fragments were performed to create final cDNA libraries for sequencing. After library construction, the concentration and insert size of the three libraries were tested using Qubit 2.0 fluorometer and Agilent 2100 Bioanalyzer, respectively. The valid concentration of the three libraries was accurately quantified with Q-PCR method to ensure reliability. The cDNA libraries were sequenced on the HiSeq2500 platform (Illumina, USA) with reads length 100 bp, and the sequencing reads were submitted to NCBI (BioProject ID: PRJNA361017; BioSample ID: SAMN06219348, SAMN06219349, SAMN06219350; SRA Accession Number: SRR5177037,  SRR5177036 and  SRR5177035).

### *De novo* assembly of transcripts and unigenes

The raw sequencing reads from transcriptome libraries were filtered with the following steps to obtain high-quality clean reads for *de novo* assembly. First, reads with adaptor sequences were removed. Then, low-quality reads, which are defined as low quality bases (*Q* < 20 or ‘N’) are more than 10%, were discarded. After filtering, the clean reads from three samples were pooled and then assembled into transcripts using Trinity (Grabherr et al., 2011) (https://github.com/trinityrnaseq/trinityrnaseq/wiki). Transcripts shorter than 300 bp were removed to eliminate redundant sequences, and the longest transcript in each cluster was selected and represented as the final unigenes.

### Characteristics of the Chinese goldthread unigenes

MIcroSAtellite identification tool (MISA) (http://pgrc.ipk-gatersleben.de/misa/) was used to identify perfect simple sequence repeats (SSRs). The requirements for each SSR types were set as followings: >10 repeats for mono-nucleotide SSRs, >6 repeats for di-nucleotide SSRs, >5 repeats for tri-nucleotide SSRs, >5 repeats for tetra-nucleotide SSRs, >5 repeats for penta-nucleotide SSRs and >5 repeats for hexa-nucleotide SSRs. SSRs with less than 100 interspace nucleotides were defined as compound SSRs. Clean reads from three samples were aligned to unigenes using SOAPaligner (Li et al., 2009a), and SOAPsnp (Li et al., 2009b) was used to identify potential SNPs (single nucleotide polymorphism) in unigenes.

### Annotation of the Chinese goldthread unigenes

Putative coding sequences (CDS) were predicted using the GetORF function in the EMBOSS software packages (Rice, Longden & Bleasby, 2000), and these CDS were translated into amino acid sequences according to the Standard Amino Acid Codon table. Unigenes were aligned to functional databases, including non-redundant (NR), SwissProt (Boeckmann et al., 2003), the Kyoto Encyclopedia of Genes and Genomes (KEGG) (Kanehisa & Goto, 2000) and the Clusters of Orthologous Groups of proteins (COG) (Tatusov et al., 2003), by carrying out a BLAST (Camacho et al., 2009) analysis using an E-value cutoff of 1e-5. The resulting NR BLASTP hits were further processed by the Blast2GO software (Conesa et al., 2005) to retrieve associated GO terms (Ashburner et al., 2000).

### Identification of differentially expressed genes between different biotopes

Bowtie (Langmead et al., 2009) was used to align clean reads of each sample to the unigene set and the RSEM program (Li & Dewey, 2011) was used to estimate gene expression levels. FPKM (Fragments Per Kilobase of transcript per Million mapped reads) was used to represent the expression levels of corresponding unigene (Trapnell et al., 2012). EBSeq (Leng et al., 2013) was used to perform differential expression analysis and to identify differential expression gene sets between samples. Benjamini–Hochberg method was used to correct *p*-value to avoid for occurrence of false positives. In our analysis, the threshold of FDR (false discovery rate) <0.01 and log2FC ≥ 2 (≥4 fold change between two samples) were set to identify differentially expressed genes (DEGs). To reveal the expression pattern of DEGs in each biotope, a hierarchical clustering, which could cluster the genes with same and similar expression level, was performed. To explore the function of DEGs, DEGs were annotated to COG (Cluster of Orthologous Groups of proteins) database (Tatusov et al., 2003) to classify reliable orthologs. DEGs were also annotated to GO databases and KEGG database and KEGG enrichment analysis of DEGs was conducted using fisher’s exact test.

**Figure 1:**
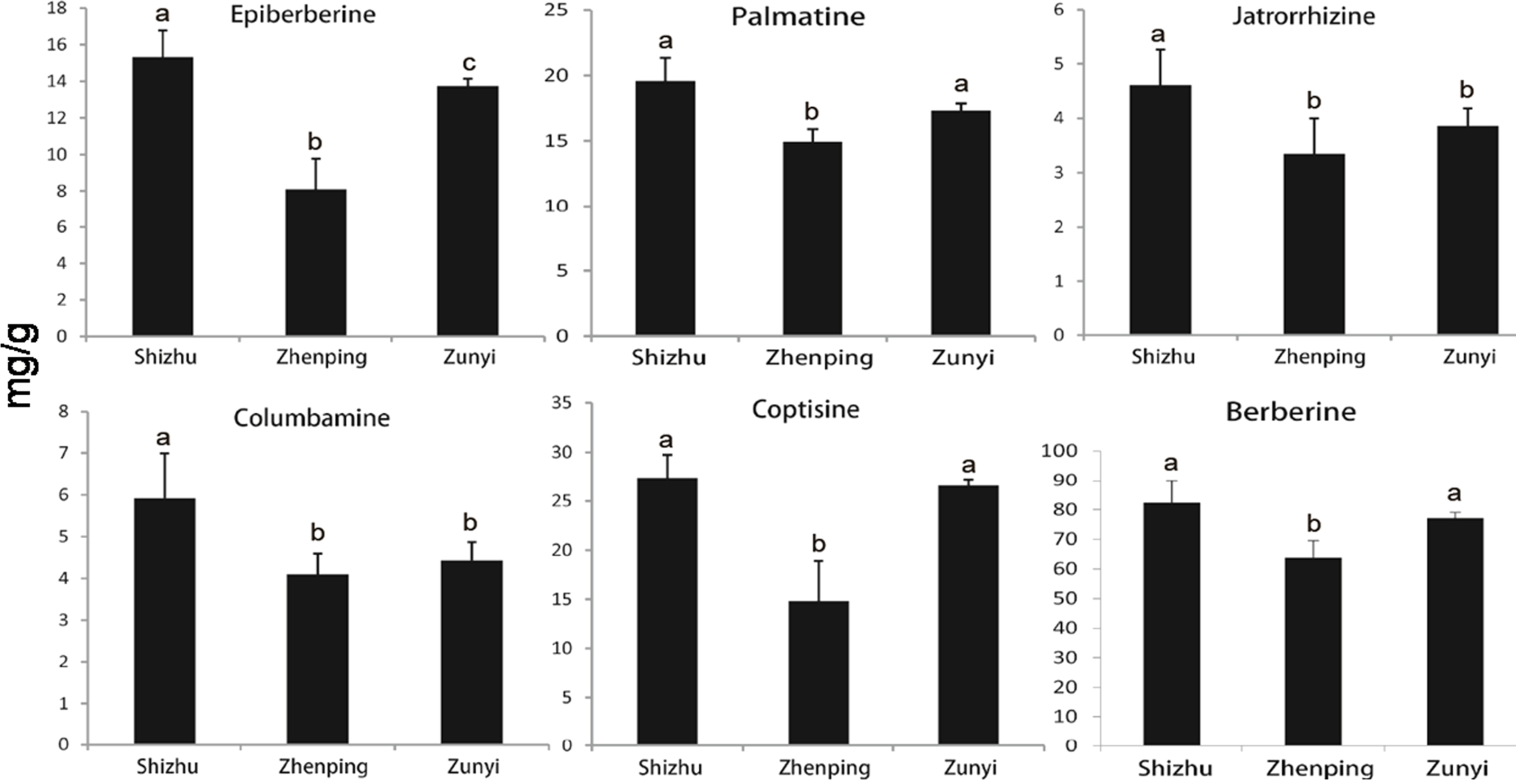
The contents of six main alkaloids in the Chinese goldthread from three biotopes. For each kind of alkaloid, statistical analysis on one-way analysis of variance (ANOVA) was performed using Microcal Origin software (version 8.0). Significant differences (*P* < 0.05) between treatments are indicated by different letters. mg/g: the milligram of alkaloid per one gram dried tissue.

## Results and Discussion

### Chemical analysis of the main alkaloids in the Chinese goldthread from three biotopes

The developed HPLC method has been proven to be an efficient method in detecting the main alkaloids of Chinese goldthread, and was applied to simultaneously analyse main alkaloids. In this study, alkaloids in the samples from Shizhu (T03), Zunyi (T02) and Zhenping (T01) were determined (Fig. 1). Totally, six major alkaloids were quantified, i.e., berberine, palmatine, coptisine, epiberberine, columbamine, and jatrorrhizine, and subsequently evaluated by comparison with authentic standards. For each sample, berberine has the highest concentrations, followed by coptisine, palmatine, and epiberberine sequentially. More interestingly, we found that the concentrations of each alkaloid and total alkaloids in sample from Shizhu are all higher than those in samples from other two biotopes. Especially, for the most abundant berberine, the concentration is 82.44 ± 7.65 mg/g in Shizhu (T03), which is higher than the value of 77.17 ± 1.87 mg/g in Zunyi (*p* > 0.05) and 63.79 ± 5.76 mg/g in Zhenping (T01) (*p* < 0.05). As the effective constituent in the Chinese goldthread, berberine has been widely used as marker of the drug quality in China (Fan et al., 2012). Therefore, we suggest that the drug quality of the Chinese goldthread from Shizhu (T03) may be the highest among the three biotopes.

### *De novo* transcriptome assembly captures high-quality transcripts and unigenes

**Figure 2:**
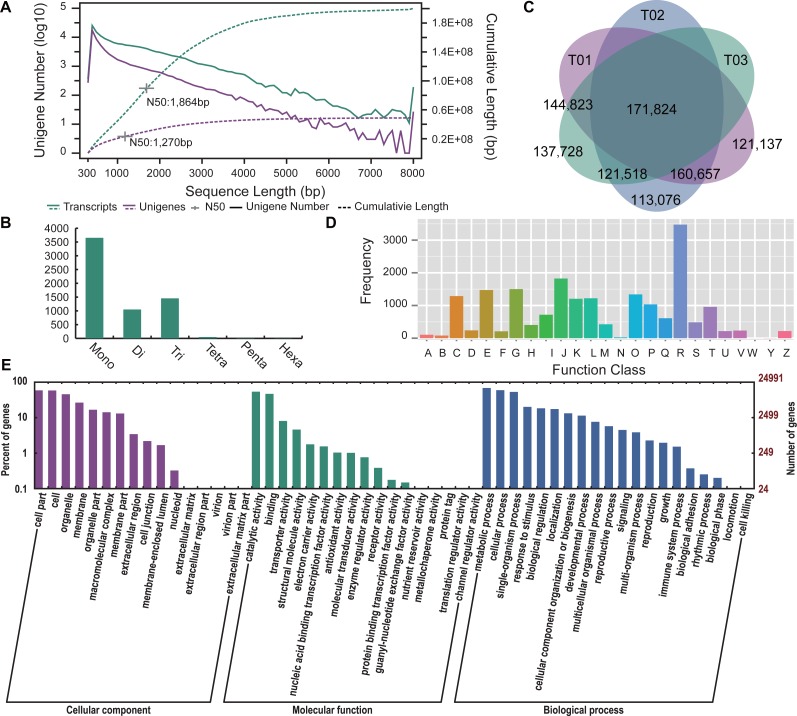
Assembly and annotation of the transcripts and unigenes of the Chinese goldthread. (A) Distribution of transcripts and unigenes length. The left *y*-axis and solid lines are the distributions of number (log10-transformed) of assemblies in each 100-bp bin, while the right *y*-axis and dashed lines are the cumulative length curves for each assembly. (B) Number distribution of SSRs identified in unigenes (length ≥ 1 kb). Mono, Mono-nucleotide; Di, Di-nucleotide; Tri, Tri-nucleotide SSRs; Tetra, Tetra-nucleotide SSRs; Penta, Penta-nucleotide SSRs; Hexa, Hexa-nucleotide SSRs. (C) Venn diagrams illustrating SNPs either shared or unique to each accession (Zhenping , T01; Zunyi, T02; Shizhu, T03). (D) COG functional classification of unigenes. (E) GO functional classification of unigenes.

To obtain a comprehensive reference unigenes of the Chinese goldthread, transcriptome of three accessions from three biotopes were sequenced using the Illumina HiSeq platform (Table 1). After the removal of adapter sequences contaminated reads, low-quality reads and duplicated reads, a total of 14.89 GB clean data was obtained, including 25,088,487, 23,905,895 and 24,723,011 pairs for Zhenping (T01), Zunyi (T02) and Shizhu (T03) respectively. These clean reads were then pooled and assembled by Trinity (k-mer length = 25) program, producing 155,710 transcripts and 56,071 unigenes (Fig. 2A, Table S1). The N50 value was 1,864bp and 1,270 bp for transcripts and unigenes respectively. To assess the quality of assembly, clean reads were mapped to the assembled unigenes. More than 81.69%, 83.24%, and 80.29% of clean reads were mapped for three samples respectively (Table 1). We further used a core eukaryotic gene mapping approach [CEGMA (Parra, Bradnam & Korf, 2007)] to identify the core genes in the Chinese goldthread transcripts and unigenes; and more than 98.39% and 95.97% of core eukaryotic genes were found in the transcripts and unigenes respectively (Table 2). These results indicated that our assembled transcripts and unigenes were excellent for downstream analyses.

**Table 1:** Statistics of the transcriptome sequencing reads data of three samples.

Collection	Sample	Read sum	Base sum	N (%)	Q30 %	Mapped reads	Mapped ratio
Zhenping, Shaanxi	T01	25,088,487	5,066,126,786	0.02	90.41	20,495,970	81.69%
Zunyi, Guizhou	T02	23,905,895	4,827,775,131	0.04	90.65	19,900,335	83.24%
Shizhu, Chongqing	T03	24,723,011	4,993,262,950	0.04	91.10	19,848,914	80.29%

**Table 2:** Statistics of the completeness of the Chinese goldthread transcripts and unigenes based on 248 CEGs using CEGMA.

Collection		Prots	Completeness	Total	Average	Ortho
transcripts	complete	238	95.97	760	3.19	78.99
	partial	244	98.39	909	3.73	86.48
unigenes	complete	232	93.55	536	2.31	67.67
	partial	238	95.97	671	2.82	78.57

**Notes.**

Prots number of 248 ultra-conserved CEGs present in genome %Completeness percentage of 248 ultra-conserved CEGs present Total total number of CEGs present including putative orthologs Average average number of orthologs per CEG %Ortho percentage of detected CEGs that have more than 1 ortholog

### Characteristics of the Chinese goldthread unigenes

Simple sequence repeats (SSRs) are tandem sequences widely distributed across the entire genome and are of high genetic diversity. Using MISA (http://pgrc.ipk-gatersleben.de/misa/), 6,224 SSRs were identified in 14,585 unigenes with length over 1kb (Fig. 2B). Approximately 31.6% (4,608 out of 14,585) of filtered unigenes have SSRs. The most prevalent SSRs type is mono-nucleotide SSRs, which is followed by tri-nucleotide SSRs. 529 SSRs presented in compound form. A total of 8.17% of the sequences have more than one SSR.

To further explore the genetic diversity in genic region in different biotopes, clean reads from three samples were mapped to unigenes to call variants (Table S2). Totally, 1,037,576 SNPs were identified in all three samples, and among of them, 970,763 are biallelic. For the three accessions from Zhenping (T01), Zunyi (T02), and Shizhu (T03), 598,441, 567,075, and 575,893 biallelic sites were identified respectively and 171,824 sites were shared by all the accessions (Fig. 2C). Only about 60% of total biallelic sites identified in each biotope and 18% of total biallelic sites identified in all the three biotope indicated a high variance among the biotopes. Moreover, 520,462, 493,718, and 507,247 heterozygous biallelic sites were identified in the three accessions from Zhenping (T01), Zunyi (T02), and Shizhu (T03) respectively (Table S2). These heterozygous sites contribute to as highly as ∼1% of total length of unigenes, indicating high polymorphism in coding regions of the Chinese goldthread. This heterozygosity rate in coding regions is comparable to the whole genome heterozygosity rate (∼1.1%) reported in a previous genome survey (Sun et al., 2014), and further demonstrated that the genome of the Chinese goldthread is highly heterozygous.

### Annotation of the Chinese goldthread unigenes

Annotation provides important information of the gene repositories. Totally, 56,028 CDSs were predicted by GetORF (Rice, Longden & Bleasby, 2000), with the total length reaching 27,544,206 bases. The N50 of the predicted CDS for unigenes was 858 bp, while the mean length was about 492 bp (Table S3). Approximately 100% of assembled unigenes were annotated with CDS confirming the high quality of the de novo assembly. Functional annotation of the assembled unigenes was obtained according to the sequence similarity to functional databases including NR, Swiss-Prot, KEGG, COG and GO. 19,274 unigenes were categorized into 25 functional COG clusters (Fig. 2D), and 24,991 unigenes were annotated to 128,452 GO terms (Fig. 2E), with 59,470 (46.30%) for biological processes, 30,102 (23.43%) for cellular components, and 38,880 (30.27%) for molecular functions. To identify biological pathways of unigene, unigenes were annotated to KEGG database and a total of 11,573 unigenes were identified in 120 KEGG pathways (Table S4). Totally, 37,100 unigenes were functionally annotated in the five databases (Table S5), making up more than 66% of total unigenes.

**Figure 3:**
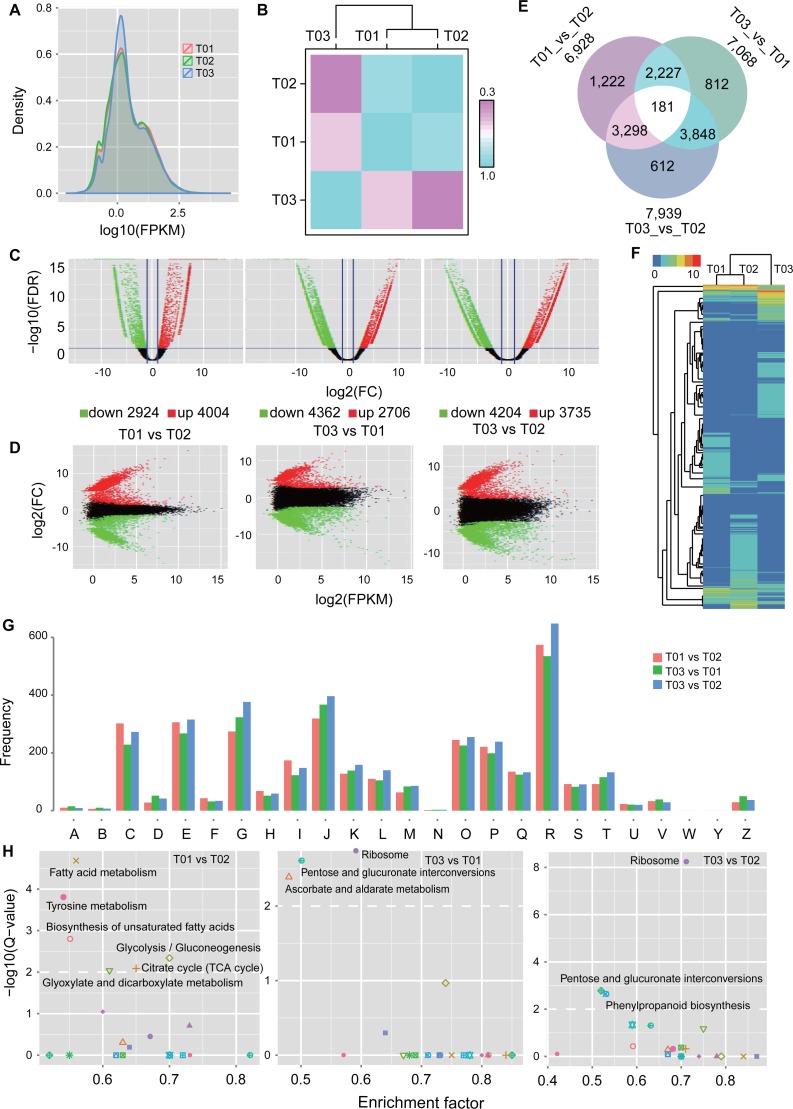
Differential expressed unigenes in the Chinese goldthread from Zhenping (T01), Zunyi (T02) and Shizhu (T03). (A) Distribution of FPKM values from each sample. (B) Pearson correlation of the samples. Each grid denotes the corresponding Pearson correlation coefficient between the two samples. (C, D) Global view of transcriptional changes by volcano and MA plot of unigenes. Genes with >2.0 log2 fold-change values and FDR < 0.01 were identified as significantly differentially expressed genes and colored with red and green. (E) Venn diagrams illustrating differentially expressed unigenes either shared or unique to specific comparison. (F) Hierarchical clustering analysis of unigene expression profiles of DEGs from the three samples. (G) COG annotation of DEGs. (H) KEGG enrichment analyses of DEGs, and significantly over-represented maps were labeled.

### Identification of differentially expressed genes among three biotopes

To identify pivotal candidate genes responsible for the difference of concentrations of alkaloids, we quantified the expression level of unigenes in the Chinese goldthread from Zhenping (T01), Zunyi (T02) and Shizhu (T03), and then differentially expressed genes (DEGs) between two biotopes were identified (Fig. 3). FPKM (Fragments Per Kilobase of transcript per Million mapped reads) (Trapnell et al., 2012) calculated by RSEM (Li & Dewey, 2011) was used to represent the expression levels of unigene. The distribution of FPKM values revealed that the expression pattern of Shizhu (T03) is different from that of Zhenping (T01) and Zunyi (T02) (Fig. 3A), and this was also evidenced by the Pearson correlation analysis (Fig. 3B), which clustered Zhenping (T01) and Zunyi (T02). To identify DEGs involves in alkaloids metabolism, three comparisons including Zhenping–Zunyi (T01 vs T02), Zhenping–Shizhu (T01 vs T03), and Zunyi–Shizhu (T02 vs T03) were performed, and the thresholds were set as FDR < 0.01 and fold change ≥ 4 (Figs. 3C and 3D). There were 6,928 DEGs identified between Zhenping (T01) and Zunyi (T02) including 4,004 up-regulated genes (57.79% of DEGs) and 2,924 down-regulated genes (42.21% of DEGs) (Table S6), 7,068 DEGs identified between Zhenping (T01) and Shizhu (T03) containing 2,706 up-regulated genes (38.29% of DEGs) and 4,362 down-regulated genes (61.71% of DEGs) (Table S7), and 7,939 DEGs identified between Zunyi (T02) and Shizhu (T03) with 3,735 up-regulated genes (47.05% of DEGs) and 4,204 down-regulated genes (52.95% of DEGs) (Table S8). 181 unigenes were differentially expressed in all the comparison among the three samples (Fig. 3E), while 12,200 unigenes were differentially expressed in at least one comparison among the three samples (Fig. 3F).

To explore the function of these DEGs, their annotation information were extracted from previous annotation results. A total of 5,614, 5,780 and 6,667 DEGs from the comparison of Zhenping (T01)–Zunyi (T02), Zhenping (T01)–Shizhu (T03), and Zunyi (T02)–Shizhu (T03) were annotated respectively (Table S9). A total of 125∼135 DEGs were related to secondary metabolites biosynthesis, transport and catabolism in each comparison (Table S10). The distribution of number of DEGs in each COG category (Fig. 3G, Table S10) and GO terms (Fig. S1) revealed global similarity, which is similar to the functional distribution of whole proteome (Fig. S1), indicating a complex difference among accessions. By mapping the DEGs to KEGG pathway (Tables S11–S13), enrichment analysis based on fisher’s exact test were performed. For the Zhenping (T01)-Shizhu (T03), the DEGs were significantly over-presented in ‘ribosome’ (corrected *p* value: 0.00E+00), ‘pentose and glucuronate interconversions’(corrected *p* value: 2.46E–03), and ‘ascorbate and aldarate metabolism’ (corrected *p* value: 4.08E–03); while for the Zunyi (T02)-Shizhu (T03), the DEGs were over-presented in ‘ribosome’ (corrected *p* value: 5.85E–09), ‘pentose and glucuronate interconversions’ (corrected *p* value: 1.67E–03), and ‘phenylpropanoid biosynthesis’ (corrected *p* value: 2.31E–03) (Fig. 3H, Table S14).

### Candidate genes involved in alkaloids biosynthesis pathways

**Figure 4:**
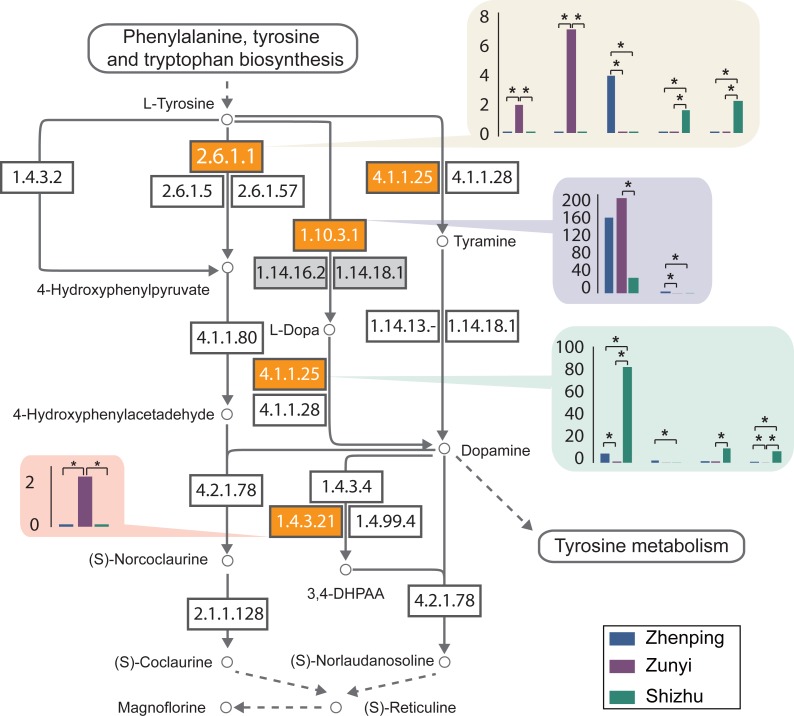
The DEGs in the isoquinoline alkaloid biosynthesis pathway. Circles indicate chemical components; rectangles are enzymes with the EC numbers given; arrows are the direction of reaction; bar plots show the FPKM values in each sample, and star marks the DEGs.

The rhizome of the Chinese goldthread contains at least six effective medicinal ingredients, including jateorhizine, columbamine, epiberberine, coptisine, palmatine and berberine (Fig. 1). Four out of the six medicinal components, including the major component berberine, palmatine, epiberberine, and columbamine, belong to isoquinoline alkaloid. Therefore, we surveyed candidate genes in the Chinese goldthread involved in the isoquinoline alkaloid biosynthesis pathway (http://www.genome.jp/kegg-bin/show_pathway?map00950). Surveying the KEGG annotation for all of the unigenes, 32 unigenes were mapped to the isoquinoline alkaloid biosynthesis pathway (Fig. S2, Table S15). Totally, 12 DEGs were identified in this pathway (Table S16). 8 DEGs were identified between Zhenping (T01) and Zunyi (T02), 8 DEGs were identified between Zhenping (T01) and Shizhu (T03), and 9 DEGs were identified between Zunyi (T02) and Shizhu (T03). These 12 DEGs only belongs to four enzymes (aspartate aminotransferaseprotein, EC: 2.6.1.1; polyphenol oxidase, EC: 1.10.3.1; primary-amine oxidase, EC: 1.4.3.21; tyrosine decarboxylase, EC: 4.1.1.25) (Fig. 4).

Isoquinoline alkaloids are tyrosine-derived plant alkaloids with an isoquinoline skeleton. The tyrosine was first transformed to reticuline, an important precursor of various benzylisoquinoline alkaloids, including analgesic compounds of morphine and codeine, and anti-infective agents of berberine, palmatine, and magnoflorine (Fig. S2). Interestingly, all the four differentially expressed enzymes located in the upstream of the biosynthesis of reticuline, indicating that the biosynthesis of reticuline may be the key regulatory steps for the active alkaloids biosynthesis. The aspartate aminotransferase (AAT, EC 2.6.1.1) catalyses the transamination between glutamate and oxaloacetate to generate 2-oxoglutarate and aspartate, the precursor for the biosynthesis of amino acids and derived metabolites. The expression levels of the five *AAT* genes are significantly different among the three accessions (Fig. 4), indicating possible different efficiency in the formation of aromatic tyrosine. The tyrosine decarboxylase (TYDC, EC: 4.1.1.25) catalyses the transformation between tyrosine and tyramine, and all the four *TYDC* genes have the highest expression level in accession Shizhu (T03) (Fig. 4), which also has the highest alkaloids concentration (Fig. 1). Therefore, the TDC is most likely to have an important function in the initial reaction of the active alkaloids biosynthesis pathway in the Chinese goldthread. Altogether, one possibility that the AAT control the formation of aromatic tyrosine and the TYDC exerts regulatory control over tyrosine through the alkaloids biosynthesis pathway.

## Conclusions

In this study, we analyzed the active metabolites and the transcriptome of the Chinese goldthread from three biotopes, including Zhenping, Zunyi and Shizhu. Chemical analyses of the rhizome indicated that berberine is the most abundant, followed by coptisine, palmatine, and epiberberine sequentially for all the three accessions. As revealed by the concentrations of active alkaloids, the drug quality of the accession from Shizhu may be the highest among these accessions. We built the high-quality transcripts and unigenes for the Chinese goldthread, and more than 98.39% and 95.97% of core eukaryotic genes were found in the transcripts and unigenes respectively, indicating that this unigene set captures the majority of the coding genes. SNPs were called in the three accessions and the results revealed high polymorphism in coding regions of the Chinese goldthread (∼1%). Differential analyses of the transcriptome identified four pivotal candidate enzymes, including aspartate aminotransferaseprotein, polyphenol oxidase, primary-amine oxidase, and tyrosine decarboxylase, were significantly differentially expressed and may be responsible for the difference of alkaloids contents in the accessions from different biotopes. In total, our study promotes the understanding of the metabolic pathways of active alkaloids components and provides substantial genetic resources for further researches.

##  Supplemental Information

10.7717/peerj.3303/supp-1 Supplemental Information 1 Supplementary data

## Additional Information and Declarations

### Data Availability

The following information was supplied regarding data availability: The sequencing reads were submitted to NCBI (SRA Accession Number: SRR5177037, SRR5177036 and SRR5177035) and we have an e-mail from NCBI to release the raw data currently. The raw data has also been uploaded as a Supplementary File.
